# Retraction: MiR-27a promotes the autophagy and apoptosis of IL-1β treated-articular chondrocytes in osteoarthritis through PI3K/AKT/mTOR signaling

**DOI:** 10.18632/aging.204906

**Published:** 2024-01-30

**Authors:** Chen Cai, Shaoxiong Min, Bo Yan, Wen Liu, Xiao Yang, Liuxun Li, Ting Wang, Anmin Jin

**Affiliations:** 1 Department of Spine Surgery, Zhujiang Hospital, Southern Medical University, Guangzhou, Guangdong, China; 2 Department of Spine Surgery, The Third Affliated Hospital of Southern Medical University, Guangzhou, Guangdong, China; 3 Department of Cell Biology, School of Basic Medical Science, Southern Medical University, Guangzhou, Guangdong, China

**Keywords:** osteoarthritis, apoptosis, autophagy, miR-27a, PI3K

**This article has been retracted:** Aging has completed its investigation of this paper. We found that four images of immunofluorescent staining in Figure 5A were previously published as Figure 6A in a 2012 paper by different authors [1]. In addition, Western blot bands in Figure 5B show internal duplication with bands in Figures 7B and Figure 2C in an earlier published paper from Huang et al [2] as well as Figure 6A in an unrelated paper [3]. There are also multiple overlaps between Western blot bands in Figures 6G and 7B with figures in unrelated papers all published in 2019 [4–6]. The authors informed the journal that they cannot reproduce the data published in the paper. As a result, all authors agreed that the article should be retracted.

## References

[1] Kumari S, Mehta SL, Li PA. Glutamate induces mitochondrial dynamic imbalance and autophagy activation: preventive effects of selenium. PLoS One. 2012; 7:e39382. 10.1371/journal.pone.0039382 22724008 PMC3378533

[2] Huang YH, Sun Y, Huang FY, Li YN, Wang CC, Mei WL, Dai HF, Tan GH, Huang C. Toxicarioside O induces protective autophagy in a sirtuin-1-dependent manner in colorectal cancer cells. Oncotarget. 2017; 8:52783–91. 10.18632/oncotarget.17189 28881770 PMC5581069

[3] Huang L, Jian Z, Gao Y, Zhou P, Zhang G, Jiang B, Lv Y. RPN2 promotes metastasis of hepatocellular carcinoma cell and inhibits autophagy via STAT3 and NF-κB pathways. Aging (Albany NY). 2019; 11:6674–90. 10.18632/aging.102167 31481647 PMC6756868

[4] Liang M, Shao A, Tang X, Feng M, Wang J, Qiu Y. MiR-34a affects dexmedetomidine-inhibited chronic inflammatory visceral pain by targeting to HDAC2. BMC Anesthesiol. 2019; 19:131. 10.1186/s12871-019-0801-z 31324142 PMC6642536

[5] Li G, Tan W, Fang Y, Wu X, Zhou W, Zhang C, Zhang Y, Liu Y, Jiu G, Liu D. circFADS2 protects LPS-treated chondrocytes from apoptosis acting as an interceptor of miR-498/mTOR cross-talking. Aging (Albany NY). 2019; 11:3348–61. 10.18632/aging.101986 31141496 PMC6555446

[6] Chen T, Zhang Y, Liu Y, Zhu D, Yu J, Li G, Sun Z, Wang W, Jiang H, Hong Z. MiR-27a promotes insulin resistance and mediates glucose metabolism by targeting PPAR-γ-mediated PI3K/AKT signaling. Aging (Albany NY). 2019; 11:7510–24. 10.18632/aging.102263 31562809 PMC6781997

